# Dr. Charles S. Payne

**Published:** 1918-02

**Authors:** 


**Dr. Charles S. Payne, Buffalo** 1884, died at his home in Liberty, Dec. 12, from cerebral haemorrhage, aged 57.

